# Prevalence and risk factors of stillbirths among pregnant women from twelve high-volume birthing facilities of Karachi, Pakistan: a longitudinal cohort study

**DOI:** 10.1186/s12884-025-08288-3

**Published:** 2025-12-30

**Authors:** Danya Arif Siddiqi, Muhammad Zia Muneer, Sundus Iftikhar, Mubarak Taighoon Shah, Vijay Kumar Dharma, Fatima Miraj, Mariam Mehmood, Irshad Ali Sodhar, Farrukh Raza Malik, Subhash Chandir

**Affiliations:** 1 IRD Global, The Great Room, Level 10, Singapore, One George Street, 049145 Singapore; 2 Department of Infectious Disease Epidemiology and Dynamics, London School of Hygiene & Tropical Medicine, London, Keppel St., WC1E 7HT UK; 3 Maternal & Child Health, IRD Pakistan, 4th Floor Woodcraft Building Korangi Creek, Karachi, 75190 Pakistan; 4 Department of Health, Government of Sindh, Karachi, Pakistan; 5 Establishment Division, Government of Pakistan, Karachi, Pakistan

**Keywords:** Stillbirths, Pregnancy outcomes, Maternal health, Birthing facilities, Pre-term births, Antenatal care

## Abstract

**Background:**

Stillbirth, defined as the death of a fetus at or after 22 weeks of gestation, remains a neglected public health issue, with approximately 2 million stillbirths occurring annually. Pakistan ranks among the top three countries with the highest number of stillbirths, yet progress in reducing stillbirth rates remains slower than regional and global averages. Despite the substantial burden, there is a lack of evidence on the prevalence, geographic variation, and predictors of stillbirths in Pakistan, particularly from marginalized settings.

**Methods:**

We conducted a longitudinal cohort study in 12 selected public and private birthing facilities located in Karachi, Sindh, Pakistan between February 9, 2021, and January 1, 2022. We enrolled pregnant women visiting the selected birthing sites during antenatal care visits and those directly visiting for deliveries. We used the World Health Organization (WHO) standard definition of stillbirth occurring at a gestational age of ≥22 weeks. We analyzed stillbirth rates across birthing sites, geographic location, and gestational age, and used firth logistic regression to identify risk factors for stillbirths.

**Results:**

Of the pregnant women enrolled (*n* = 21,523), 63.5% (13,668/21,523) with a gestational age ≥ 22 weeks delivered their babies at the study birthing facilities. The overall weighted stillbirth rate was 12.0 per 1,000 births across all sites. The prevalence varied substantially across sites, geographic location, gestational age, and facility type (public or private). Multivariable logistic regression showed a significant association between polio-endemic super high-risk union councils (AHR: 3.53; CI: 1.84–6.75), preterm delivery (AHR: 3.97; CI: 1.42–11.16), unvaccinated for Tetanus Toxoid (TT) vaccine during pregnancy (AHR: 5.29; CI: 2.61–10.74), and having received <8 ANC visits (AHR: 2.40; CI: 1.04–5.53) with stillbirth outcomes.

**Conclusion:**

Our study found significant variation in stillbirth prevalence across birthing facilities and geographic locations, with notably higher stillbirth rates in polio-endemic regions. These findings highlight the need for integrated approaches that combine polio eradication efforts with enhanced maternal healthcare services including maternal immunizations to maximize efficiency and impact. Additionally, efforts are needed to ensure high-quality antenatal care services and efficient management of medical conditions and prematurity during pregnancy.

**Supplementary Information:**

The online version contains supplementary material available at 10.1186/s12884-025-08288-3.

## Background

Stillbirths are defined as the death of a fetus at or after 22 weeks of gestation before or during delivery [1]. Despite stillbirths being an important indicator of maternal and child health, the progress achieved in reducing maternal and child mortality has not been paralleled by equivalent advancements in addressing the burden of stillbirths [2]. While the UN Global Strategy for Women’s, Children’s and Adolescents’ Health (2016–2030) and the Every Newborn Action Plan (ENAP) advocate for an end to preventable stillbirths, reducing stillbirths is conspicuously absent as a specific target in the Sustainable Development Goals agenda [3, 4]. This neglect becomes particularly concerning as the burden of stillbirths continues to rise, with approximately 2 million stillborn babies each year, with devastating and far-reaching consequences for families, communities, and health systems [5]. Prevalence of stillbirths is disproportionately higher in low- and middle-income countries (LMICs), where the risk of stillbirth is more than seven times higher than in high-income settings [6]. Over 70% of the global stillbirths occur in Sub-Saharan Africa and South Asia and in 2021, six countries – India, Pakistan, Nigeria, the Democratic Republic of Congo, Ethiopia, and Bangladesh – alone accounted for nearly half of all global stillbirths [5, 7]. With the last standardized global assessment of stillbirth rates conducted in 2019, stillbirths remain underreported or omitted from global data tracking, making it difficult to measure their true burden [2, 5, 7]. If current trends persist, over 16 million additional babies are projected to be stillborn by 2030, with 56 countries likely to miss the ENAP target of reducing stillbirths to 12 or fewer per 1,000 total births [7].

Pakistan is among the 56 countries at risk of missing the ENAP stillbirth target by 2030 [5, 7]. The country also ranks third in the number of stillbirths (> 200,000) and second in stillbirth rates (30.9 per 1000 births) [7]. Pakistan’s progress in reducing rates of stillbirths in the past two decades has been lower (1%) compared to both the regional average (3%) and other low-income countries (1.3%) [7, 8]. Factors contributing to high rates of stillbirths include socioeconomic inequalities, inadequate access to skilled birth attendants and early screening for complications, suboptimal uptake of technological advancements in healthcare and preventable maternal risk factors such as malnutrition, infections, hypertensive disorders, and diabetes [8–10]. These are compounded by systemic barriers, including under-resourced healthcare systems, weak referral mechanisms, and cultural norms that delay care-seeking behavior [11, 12].

Data on stillbirth prevalence in Pakistan remain fragmented and inconsistent, primarily due to underreporting, the absence of standardized definitions, and infrequent monitoring [8, 12]. The lack of civil and vital registration systems, and comprehensive medical birth and death registries, further complicates the production of reliable and timely stillbirth estimates [2]. Local studies report varying stillbirth rates (6 to 44.4 per 1,000 total births), but these are outdated, limited in scope, typically facility-specific, and fail to capture broader systemic patterns and regional disparities [8, 11, 13, 14]. The impact of geographical disparities, particularly district-level variations and challenges faced by polio-endemic regions, remains underexplored, despite evidence that healthcare access in these areas is often limited. Existing research also often overlooks the need to distinguish stillbirth rates by gestational age, which obscures the differing risk factors and causes of stillbirths at various stages of pregnancy. These gaps in current literature and data systems hinder the identification of key risk factors and the development of effective strategies to reduce stillbirth rates in the country.

We leveraged data from an electronic Pregnant Women and Birth Registry (PWBR) to conduct a facility-based longitudinal cohort research study at selected birthing facilities in Karachi, Pakistan. We estimated the prevalence of stillbirths, analyzed stillbirth rates across different types of birthing facilities, assessed geographic variations, and identified sociodemographic, pregnancy-related, and maternal factors associated with both early and late gestational stillbirths.

## Methods

### Study site and population

The study was conducted in the city of Karachi in Sindh province, Pakistan. With a population of over 20 million, Karachi is one of the six global megacities and the largest city in Pakistan [15]. Karachi is divided into seven districts comprising 24 towns, subdivided into union councils (UCs – the smallest geographic administrative unit). Eight of the 40 Super High-Risk Union Councils (SHRUCs) in Pakistan are located in Karachi. These UCs are classified as super high risk because of the presence of positive environmental polio cases, making them disease reservoirs with poor overall health outcomes [16]. The SHRUCs, located in four districts of Karachi are home to >1.6 million people with an approximate annual birth cohort of 60,000 live births. Karachi was selected for this study due to its status as the most linguistically and ethnically diverse urban center in Pakistan, with the largest migrant population [17]. The inclusion of SHRUCs was critical to understanding disparities in stillbirth rates, in areas with poor health infrastructure.

We conducted our study in 12 public and private birthing sites located across six of the seven districts of Karachi: four in Karachi East, three in Karachi West, two in Malir, and one each in Kemari, Karachi South, and Korangi districts. Of the 12 study sites, nine were in SHRUCs, and the remaining three were in non-SHRUCs. Purposive sampling was used for all facility selection. We first compiled a list of high-volume birthing facilities and identified those located within SHRUCs. Among facilities located outside of SHRUCs, we selected high-volume tertiary care hospitals that serve Karachi, including the SHRUC regions. Data was collected from February 9, 2021 to January 1, 2022, with varying enrollment dates across study sites. 

### Participant recruitment and data collection

We deployed an electronic Pregnant Women and Birth Registry (PWBR) in selected birthing facilities from February 2021 to January 2022 to register all pregnant women and newborn children from that facility into the registry as a routine activity. Female field workers were stationed 24/7 at each birthing facility and worked closely with the facility-based birthing staff to register all pregnant women and newborn children from that facility into the PWBR. The registration process included entering the biodata (name, address, contact number, basic demographic variables) of pregnant women and their newborns and assigning them a QR code as a unique identifier. This QR code served as the primary identifier for linking records across visits. In cases where the QR card was unavailable at follow-up, alternative identifiers such as name, CNIC number, phone number, and address were used to retrieve and update the corresponding record. This registry enrolled all pregnant women and their newborns into a centralized database connected to the Government of Sindh’s Electronic Immunization Registry (SEIR) [18].

### Data collection and procedure

Following their registration into the PWBR, a formal consent was taken from pregnant women to participate in a research study, which would involve additional data collection from them and their newborns at enrolment and during their follow-up visits. The data collection tool was developed after a thorough review of literature and adapting questions to fit the local context. A structured questionnaire was developed that collected data on household demographics, medical background of the pregnant women including underlying conditions, healthcare attendance, and use of medications, as well as information more specifically related to their pregnancy, such as frequency of ANC visits and uptake of vaccinations. If the pregnant women were unsure about particular questions (such as expected date of delivery, gestational age, date for upcoming ANC visit), data collectors were instructed to retrieve these details from the doctor or hospital patient records. Stillbirth occurrences were identified by the facility birthing staff in the obstetrics and gynecology ward.

Data was collected by trained female field workers who were supervised by field supervisors who remained in close contact with the birthing facility staff. Data was collected both during the women’s first visit to the facility as well as on subsequent routine visits, where the field workers followed up with enrolled women to record pregnancy-related health conditions and pregnancy outcomes. Responses were recorded using an Open Data Kit (ODK) application, which incorporated built-in validation constraints, skip logics and range checks to minimize entry errors at the point of data capture. Field supervisors and the study manager monitored data collection through daily field reports to track progress and identify any immediate technical or operational issues.

### Inclusion and exclusion criteria

All pregnant women presenting to the selected birthing facilities during the study period who provided written informed consent were eligible for registration. No exclusion criteria were applied for participant enrollment.

### Data quality measures

To ensure the quality of the questionnaire, the English version was translated into the local language by a team member fluent in the language and with sufficient experience of the local context. Field staff were provided with training on the study objectives, research ethics and the questionnaire. A pilot was done across four birthing facilities to evaluate the content, assess staff comprehension, and identify areas requiring modification. Based on pilot feedback, minor refinements were made to the training content to enhance clarity on key definitions and field procedures. Data quality checks were conducted by the field supervisor and study manager through daily field reports.

### Outcome variable and data analysis

The primary outcome variable was stillbirth, defined as a fetal death at ≥22 weeks of gestation as per the WHO/International Classification of Diseases (ICD-11) definition, among mothers of reproductive age [19]. We only included deliveries at ≥22 weeks of reported gestational age. Based on reported gestational age by participants, stillbirths were categorized as extremely preterm (< 28 weeks), very preterm (28–31 weeks), moderate to late preterm (32–36 weeks), at term (37–41 weeks), and post-term (≥ 42 weeks) [20, 21]. The stillbirth rate was defined as the number of stillbirths per 1,000 births among the study participants.

A two-pronged analytical approach was undertaken to address potential bias due to loss to follow-up (LTF), comprising (1) a complete-case (CC) analysis using unweighted Firth logistic regression, and (2) a LTF-adjusted analysis using inverse probability weighting (IPW) [22, 23]. Firth’s penalized likelihood logistic regression was selected for the unweighted CC analysis due to its suitability in addressing small sample sizes and rare binary outcomes as it prevents infinite estimates in models with rare events, making it well-suited for sparse data and low event rates [24].

For the LTF-adjusted analysis, a sensitivity assessment was conducted, and IPW was applied to account for differential follow-up probability. To mitigate the influence of extreme or unstable weights, often driven by unmeasured confounding, weights exceeding the 99th percentile were truncated to the 99th percentile value.

The weighted analysis was performed using Cox proportional hazards regression with the svy package to incorporate IPW. IPW Cox regression is particularly suitable for handling informative censoring and producing unbiased hazard ratio estimates under incomplete follow-ups [25, 26]. A manual stepwise approach was used for variable selection in the final multivariable model. An a priori list of variables was developed based on prior literature, biological plausibility, and their role as potential confounders. Variables with a p-value < 0.20 in univariable analysis were considered for inclusion, and those were retained if statistically significant (*p* < 0.05) or if they had a meaningful influence on other effect estimates. Model selection was further guided by minimizing the Akaike Information Criterion (AIC). Statistical significance was set at a two-sided p-value of < 0.05.

Multicollinearity among variables was assessed using variance inflation factors (VIFs), all of which were below the commonly accepted threshold of 5, indicating no significant multicollinearity. In addition, a correlation heatplot was examined to visually assess inter-variable relationships. Because model building was conducted manually, VIFs were reassessed at each step of adding or removing variables to ensure stability.

Analysis was conducted using Stata version 17.0. Summary measures, frequencies, and percentages were reported for categorical variables. Where relevant, both unweighted and weighted estimates were presented to reflect the underlying population structure.

## Results

### Stillbirth prevalence

**Fig. 1:**
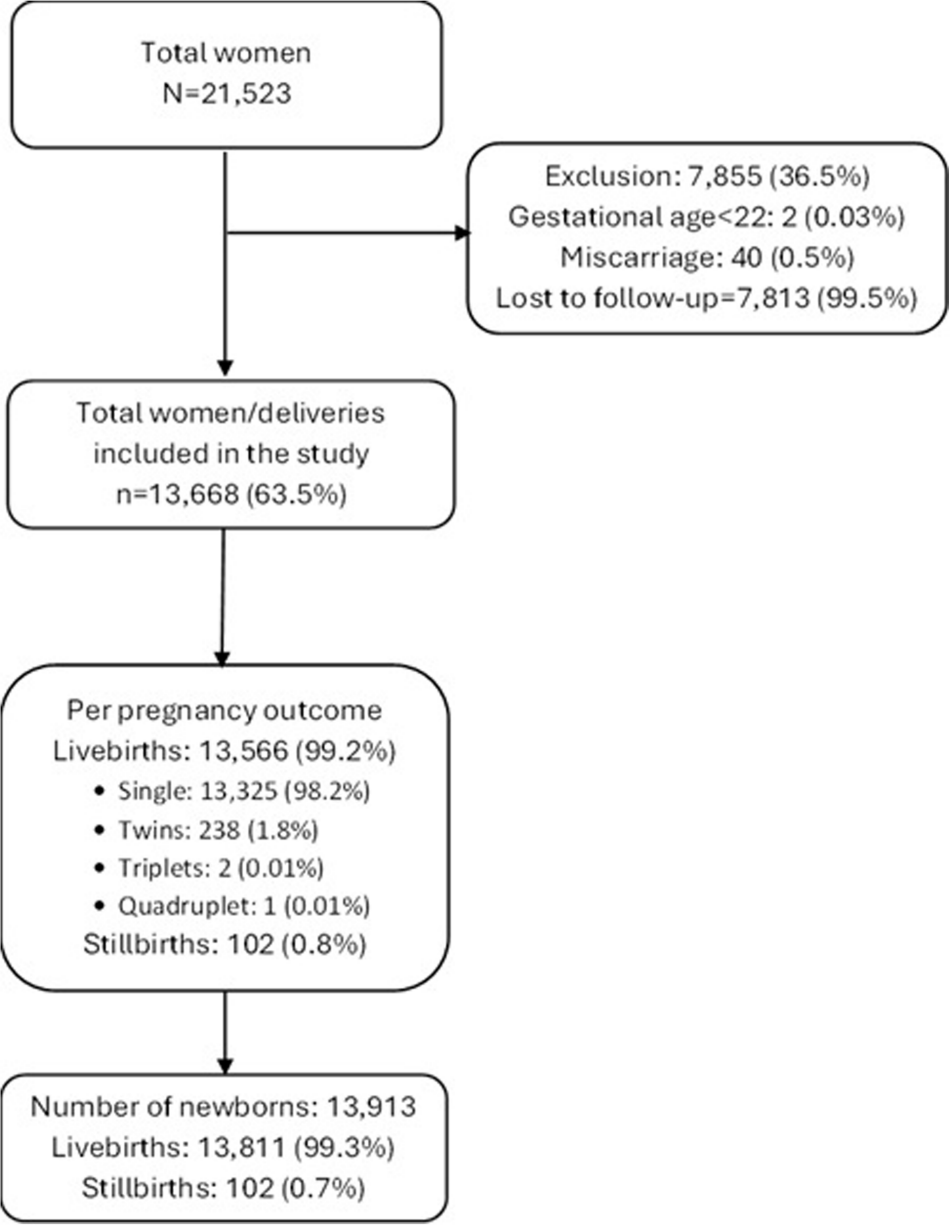
Flow diagram of participants included in the study

Between February 9, 2021, to January 1, 2022, 21,523 pregnant women enrolled in the research study. Of these, 63.5% (13,668/21,523) pregnant women with a gestational age of ≥22 weeks delivered their babies at the study birthing facilities during the study period (Fig. 1) to while, 33.5% (7,813/21,523) were lost to follow up. Supplementary Table-1 presents the baseline characteristics of women who were lost to follow-up compared to those who were retained for analysis. We recorded 102 stillbirths and 13,811 live births (including multiple births, e.g., twins or triplets, etc.), resulting in a total weighted stillbirth rate of 12.0 per 1000 births (95 CI%: 7.7–16.3) across the 12 birthing facilities. When observed on a granular level, the weighted stillbirth rate varied widely, ranging from as low as 1.3 to as high as 66.0 per 1,000 births. Higher weighted stillbirth rates were observed in SHRUCs (24.1 per 1,000 births; 95% CI: 11.9–36.3) compared to non-SHRUCs (5.6 per 1,000 births; 95% CI: 4.2–7.0.2.0), and in private facilities (17.9 per 1,000 births; 95% CI: 4.2–31.6) compared to public ones (9.6 per 1,000 births; 95% CI: 7.2–12.0) reflecting substantial disparities across facilities, its type, and location. When analyzed by gestational age, the highest weighted stillbirth rates was observed among extreme preterm births (< 28 weeks) at 429.1 per 1000 births (95% CI: 62.9–795.3), followed by 187.3 (95% CI: 0.0–437.2) among very preterm births (28–31 weeks) (Table 1).

**Table 1:** Prevalence of stillbirth rates (SBR) per 1000 births by type and location of birthing facilities (February 9, 2021 - January 1, 2022)

	# of birthing facilities (*n* = 12)	# of Deliveries (*n* = 13,668)	# of births (*n* = 13,913)	# of Live births (*n* = 13,811)	# of Stillbirths (*n* = 102)	Unweighted	95% CI	Weighted	95% CI
						SBR per 1,000 births		SBR per 1,000 births	
Overall	12	13,668	13,913	13,811	102	7.3	5.9–8.7	12.0	7.7–16.3
Birthing facilities ^a^									
Site A	-	41	43	42	1	23.3	0.0–68.3	23.9	0.0–71.5
Site B	-	80	85	83	2	23.5	0.0–55.8	34.6	0.0–83.8
Site C	-	41	44	41	3	68.2	0.0–142.7	66.0	0.0–146.5
Site D	-	4,176	4,305	4,290	15	3.5	1.7–5.2	3.4	1.7–5.2
Site E	-	368	385	374	11	28.6	11.9–45.2	28.8	11.3–46.2
Site F	-	92	93	92	1	10.8	0.0–31.7	8.4	0.0–25.1
Site G	-	231	235	232	3	12.8	0.0–27.1	12.0	0.0–25.6
Site H	-	65	68	65	3	44.1	0.0–92.9	43.0	0.0–94.6
Site I	-	20	22	21	1	45.5	0.0–132.5	64.3	0.0–193.8
Site J	-	1,913	1,958	1,939	19	9.7	5.4–14.0	9.6	5.2–13.9
Site K	-	3,558	3,637	3,598	39	10.7	7.4–14.1	11.8	8.1–15.4
Site L		2,981	3,038	3,034	4	1.3	0.0-2.6.0	1.3	0.03–2.6
Type of birthing facilities									
Government	5	10,192	10,378	10,293	85	8.2	6.5–9.9	9.6	7.2–12.0
Private	6	3,476	3,535	3,518	17	4.8	2.5–7.1	17.9	4.2–31.6
Birthing facilities in Super High-Risk Union Councils (SHRUCs) - Characterized by Polio endemic areas^b^									
SHRUCs	9	2,895	2,933	2,889	44	15.0	10.6–19.4	24.1	11.9–36.3
Non-SHRUCs	3	10,773	10,980	10,922	58	5.3	3.9–6.6	5.6	4.2–7.0
District of birthing facility									
Karachi Keamari	1	44	44	41	3	68.2	0.0–142.7	66.0	0–146
Karachi East	4	575	585	570	15	25.6	12.8–38.4	33.0	9.1–60.0
Karachi West	3	3,873	3,915	3,872	43	11.0	7.7–14.2	12.3	8.2–16.3
Karachi Malir	2	2,000	2,026	2,004	22	10.9	6.3–15.4	11.6	6.5–16.6
Karachi South	1	4,191	4,305	4,290	15	3.5	1.7–5.2	3.4	1.7–5.2
Karachi Korangi	1	2,985	3,038	3,034	4	1.3	0.0–2.6	1.3	0.03–2.6
Gestational age									
Extreme preterm (22–28 weeks)	-	26	27	18	9	333.3	155.5–511.1	429.1	62.9–795.3
Very preterm (28–31 weeks)	-	130	145	134	11	75.9	32.8–119.0	187.3	0.0–437.2
Moderate to late preterm (32–36 weeks)	-	2,846	2,934	2,894	40	13.6	9.4–17.8	16.3	10.2–22.4
Term and post-term (≥ 37 weeks)	-	10,666	10,807	10,765	42	3.9	2.7–5.1	8.0	3.7–12.2

a. Birthing facility names have been redacted due to confidentiality

b. National and Provincial Emergency Operations Centers (NEOC and PEOCs) identified 40 SHRUCs in Pakistan based on the presence of Wild Polio Virus (WPV). Of these 40 SHRUCs, 8 belonged to the 4/7 districts of Karachi division, while others were from the remaining provinces of Pakistan. Of the 12 study birthing facilities, 9 were located in SHRUCs

### Sociodemographic, maternal, and newborn characteristics

Table 2 presents the sociodemographic, maternal, and neonatal characteristics associated with unique births (live births and stillbirths), defined as one record per pregnancy, regardless of whether a woman delivered single or multiple newborns. This differs from Table 1, which reports aggregate birth outcomes.

**Table 2:** Sociodemographic characteristics, reported antenatal care (ANC) practices, and newborn health characteristics for unique births in 12 birthing facilities of Karachi, Sindh (*n* = 13,668) by birth status (February 9, 2021 - January 1, 2022)

Sociodemographic characteristics	Live Births (*n* = 13,566)			Stillbirths (*n* = 102)			Total (*n* = 13,668)		
	Median	IQR		Median	IQR		Median	IQR	
	Unweighted	Unweighted	Weighted	Unweighted	Unweighted	Weighted	Unweighted	Unweighted	Weighted
Woman age at enrollment (in years)	25.4	22.3–30.4	-	25.4	23.3–29.4	-	25.4	22.3–30.4	-
# of household members	6	5.0–9.0	-	6	5.0–10.0	-	6	5.0–9.0	-
# of children < 5 years	2	1.0–2.0	-	1	0.0–2.0	-	2	1.0–2.0	-
# of elderly aged > 65 years	0	0.0–1.0	-	0	0.0–1.0	-	0	0.0–1.0	-
# of children aged 5–18 years	1	0.0–2.0	-	1	0.0–2.0	-	1	0.0–2.0	-
Highest years of education received by household members	9	0.0–10.0	-	6	0.0–10.0	-	9	0.0–10.0	-
Weight of women (Kg) at last visit	65	60.0–70.0	-	65	60.0–70.0	-	65	60.0–70.0	-
	n	%		n	%		n	%	
Districts of birthing facilities	Unweighted	Unweighted	Weighted	Unweighted	Unweighted	Weighted	Unweighted	Unweighted	Weighted
Karachi East	560	4.1	16.9	15	14.7	46.8	575	4.2	17.29
Karachi Keamari	41	0.3	1.0	3	2.9	5.6	44	0.3	1.09
Karachi Korangi	2,981	22	14.3	4	3.9	1.5	2,985	21.8	14.15
Karachi Malir	1,978	14.6	11.6	22	21.6	11.1	2,000	14.6	11.6
Karachi South	4,176	30.8	30.1	15	14.7	8.6	4,191	30.7	29.84
Karachi West	3,830	28.2	26.0	43	42.2	26.4	3,873	28.3	26.04
Type of birthing facility									
Government	10,107	74.5	70.9	85	83.3	56.6	10,192	74.6	70.8
Private	3,459	25.5	29.1	17	16.7	43.4	3,476	25.4	29.2
Non-SHRUCs	10,715	78.9	65.3	58	56.9	30.5	10,773	78.8	64.8
SHRUCs	2,851	20.1	34.7	44	43.1	69.5	2,895	21.2	35.2
Participant ethnicity									
Urdu speaking muhajirs [27]	6,762	49.8	41.0	42	41.2	22.9	6,804	49.8	40.8
Sindhi	815	6	5.8	6	5.9	4.9	821	6	5.8
Punjabi	691	5.1	5.3	3	2.9	2.1	694	5.1	5.2
Pathan	3,521	26	33.5	41	40.2	62.9	3,562	26.1	33.9
Balochi	604	4.5	4.4	3	2.9	1.6	607	4.4	4.4
Others	1,173	4.5	10.0	7	2.9	5.6	1,180	4.4	9.9
Participant education (in Years)									
0	4,962	36.6	46.0	53	52	73.8	5,015	36.7	46.4
1–5	1,507	11.1	10.6	9	8.8	5.2	1,516	11.1	10.6
6–8	1,678	12.4	10.8	13	12.7	7.9	1,691	12.4	10.7
9–10	3,452	25.4	21.1	20	19.6	10.0	3,472	25.4	21.0
≥11	1,967	14.5	11.5	7	6.9	3.1	1,974	14.4	11.4
Participant current occupation									
Housewife	13,399	98.8		102	100		13,501	98.8	
Employed	123	0.9		-			123	0.9	
Unemployed	44	0.3		-			44	0.3	
Monthly Household Income (PKR)									
< 1,000 to 9,999	324	2.4	3.1	6	5.9	5.6	330	2.4	3.1
10,000 to 19,999	5,024	37	34.2	35	34.3	29.3	5,059	37	34.1
20,000 to 49,999	7,153	52.7	55.1	55	53.9	60.3	7,208	52.7	55.1
≥50,000	1,065	7.9	7.6	6	5.9	4.8	1,071	7.8	7.6
Reported # of ANC visits									
< 8	7.926	61.5	68.8	76	74.5	85.3	8,002	61.6	69.0
≥8	4,971	38.5	31.2	26	25.5	14.8	4,997	38.4	31.0
History of any TT vaccine received									
No	4,829	35.6	44.7	48	47.1	70.3	4,877	35.7	45.0
Yes	8,737	64.4	55.3	54	52.9	29.7	8,791	64.3	29.7
Use of any contraceptives ^a^									
No	12,005	88.5	90.5	87	85.3	91.4	12,092	88.5	90.5
Yes	1,561	11.5	9.5	15	14.7	8.6	1,576	11.5	9.5
Any pre-existing medical condition before ANC visit ^b^									
No	11,966	88.2	90.3	87	85.3	92.3	12,053	88.2	90.3
Yes	1,600	11.8	9.7	15	14.7	7.7	1,615	11.8	9.7
Any medical condition during ANC visit ^c^									
No	9,945	73.3	72.8	61	59.8	53.9	10,006	73.2	72.6
Yes	3,621	26.7	27.2	41	40.2	46.1	3,662	26.8	27.4
Any medications intake in pregnancy ^d^									
No	5,980	44.1	43.2	37	36.3	43.9	6,017	44.0	43.2
Yes	7,586	55.9	56.8	65	63.7	56.1	7,651	56.0	56.8
Any supplement intake in pregnancy ^e^									
No	1,540	11.4	13.0	10	9.8	5.4	1,550	11.3	12.9
Yes	12,026	88.6	87.0	92	90.2	94.6	12,118	88.7	87.1
Use of any substances in pregnancy ^f^									
No	12,318	90.8	92.1	95	93.1	96.4	12,413	90.8	92.1
Yes	1,248	9.2	7.9	7	6.9	3.6	1,255	9.2	7.9
Practicing any precautions in pregnancy ^g^									
No	3,258	24.0	27.3	26	25.5	22.8	3,284	24	27.2
Yes	10,308	76.0	72.7	76	74.5	77.2	10,384	76	72.8
Postpartum condition ^h^									
No	13,497	99.5	97.9	99	97.1	85.3	13,596	99.5	97.7
Yes	83	0.6	2.1	5	4.9	14.7	88	0.6	2.3
Body Mass Index (BMI) category ^i^									
Underweight	94	0.7	1.4	1	1	1.6	95	0.7	1.4
Normal	3,032	22.3	33.9	19	18.6	33.9	3,051	22.3	33.9
Overweight	3,963	29.2	42.9	30	29.4	46.3	3,993	29.2	42.9
Obese	2,038	15	21.8	11	10.8	18.5	2,049	15	21.8
Delivery by pregnancy weeks									
Extreme Preterm (22–28 weeks)	17	< 0.1	0.2	9	8.8	5.5	26	0.2	0.2
Very preterm (28–31 weeks)	119	0.9	0.9	11	10.8	15.3	130	1.0	1.1
Moderate to late Preterm (32–36 weeks)	2,806	20.7	19.6	40	39.2	26.9	2,846	20.8	19.7
Term (37–41 weeks)	10,597	78.1	79.1	42	41.2	52.3	10,639	77.8	78.8
Post-term (> 41 weeks)	27	0.2	0.2	0	-		27	0.2	0.2
Teenage Pregnancy (< 20 years)									
No	12,620	93.0	91.9	99	97.1	98.3	12,719	93.1	92.0
Yes	946	7.0	8.1	3	2.9	1.7	949	6.9	8.0
Newborn delivered by									
Doctor	12,904	95.1	92.9	87	85.3	80.1	12,991	95	92.8
Midwife/lady health worker/relative others	662	4.9	7.1	15	14.7	19.9	677	5	7.2

Note: Each record represents a unique birth (one per pregnancy). Analyses from this table onward are based on individual-level characteristics and exclude duplicate counts from multiple births (e.g., twins).”

a. Contraceptives include condoms, implants, injections, oral pills, and surgical procedures

b. Pre-existing medical condition before ANC visit includes any one of the key reported conditions anemia, hypertension, obesity, diabetes, chronic heart/liver/kidney diseases among others

c. Medical conditions during ANC visit include any one of the key reported conditions including fatigue, reflux, lower back/neck pain, nausea/vomiting pelvic pain, and anemia among others

d. Medication intake in pregnancy includes the use of any one of the key medications including painkillers, fever medicines, antibiotics, and anti-allergies, among others

e. Supplements intake in pregnancy includes the use of any one of the key supplements including folic acid, iron/vitamin D, and calcium, among others

f. Substance use in pregnancy includes any one of the substances including beetal nuts, prescription drugs for medical/non-medical use, tobacco, and alcohol

g. Practicing any precautions in pregnancy includes any one of the precautions including washing hands more frequently than usual, washing food before eating, avoiding raw food, resting more than usual, and avoiding contact with animals among others

h. Postpartum condition includes any of the conditions including admission to intensive care unit, pelvic floor disorder, postpartum hemorrhage, among others

i. BMI was not available for 4,480 participants

The median maternal age was 25.4 years across both groups, with an interquartile range (IQR) of 22.3–30.4 years for live births and 23.3–29.4 years for stillbirths. The median maternal education level among live births was 9.0 years (IQR: 0.0–10.0), compared to 6.0 years (IQR: 0.0–10.0) among stillbirths. Among the stillbirths, 83.3% occurred at government facilities compared to 16.7% at private facilities. Additionally, 69.5% of the stillbirths occurred in SHRUCs, compared to 30.5% in non-SHRUCs. Not Receiving the TT vaccine was reported in 70.3% of stillbirths, while 29.7% were among those who received the vaccine. Women with stillbirths also had a higher proportion reporting postpartum conditions compared to those with live births (14.7% vs. 2.1%).

The distribution of stillbirths varied across districts (highest in Karachi East: 46.8% vs. lowest in Karachi Korangi: 1.5%) and ethnicities (highest among Urdu speaking muhajirs: 42.2% vs. lowest among Balochi: 2.9%) (Table 2). Over nine in ten stillbirths (91.4%) occurred among women who did not use any contraceptive method, and just over half (52.3%) were preterm births (< 37 weeks gestation).

### Factors associated with stillbirths

Supplementary Table 2 shows the results of weighted and unweighted univariable regression models to determine predictors of stillbirths. The weighted univariable regression showed significant variation in stillbirth by district, type of birthing facilities, and location of birthing facilities. The risk of stillbirths was 2.57 times higher if the delivery took place at a government facility compared to a private birthing facility (HR: 2.57; CI: 0.97–6.81, p: 0.058). Similarly, we found a significantly higher risk of stillbirths in the SHRUCs facilities relative to non-SHRUCs (HR: 5.73; CI: 2.63–12.51, *p* < 0.001). Women having no schooling (HR: 4.27; CI: 1.59–11.46, p: 0.004), and belonging to a PKR 20 to < 50k income group (HR: 4.29; CI: 1.10–16.76 p:0.036) were associated with higher risk of having stillbirths. Maternal and pregnancy-related variables including not receiving TT vaccination (HR: 6.69; CI: 3.03–14.78; *p* < 0.001), and preterm delivery (HR: 4.52; CI: 1.57–13.04; *p* = 0.005) also led to a higher risk of stillbirth outcome, relative to the respective reference group.

In the weighted multivariable regression (Table 3), we found the risk of stillbirth was 3.53 times higher among women delivering in facilities located in SHRUCs (AHR: 3.53; CI: 1.84–6.75; *p* < 0.001), compared to non-SHRUCs birthing facilities. The odds of stillbirth decreased by 30% with each additional year of education of the household (AOR: 0.70; 95% CI: 0.55–0.89; *p* = 0.003). There was 5.29 times higher risk of having stillbirths among women who did not receive TT vaccination in the last pregnancy (AHR: 5.29; CI: 2.61–10.74; *p* < 0.001) relative to those who received a TT dose. Moreover, women who received < 8 ANC visits had 2.40 times higher risk of stillbirths (AHR: 2.40; CI: 1.04–5.53; *p* < 0.040) compared to those who received ≥8 ANC visits. Preterm delivery (< 37 weeks) was also found to be a significant predictor of stillbirth (AHR: 3.97; 95% CI: 1.42–11.16; *p* = 0.009).

**Table 3:** Unweighted and weighted multivariable regression analysis for risk factors associated with stillbirths among women delivering at 12 selected birthing facilities in Karachi, Sindh (*n* = 13,668) (February 9, 2021 - January 1, 2022)

	**Unweighted multivariable regression**			**Weighted multivariable regression**				
	**AOR**	**P-value**	**95% CI**		**AHR**	**P-value**	**95% CI**	
Birthing facilities in Super High-Risk Union Councils (SHRUCs) - Characterized by Polio endemic								
No	1				1			
Yes	2.55	<0.001	1.69	3.83	3.53	<0.001	1.84	6.75
# of household members	1.1	<0.001	1.05					
# of children <5 years	0.51	<0.001	0.40					
# of children aged 5–18 years	1.12	0.061	0.99					
Highest years of education received by household members					0.70	0.003	0.55	0.89
Reported # of ANC visits								
>=8					1			
<8					2.40	0.040	1.04	5.53
History of any TT vaccine received								
Yes					1			
No	1.74	0.008	1.16		5.29	<0.001	2.61	10.74
Preterm delivery (<37 weeks)								
No	1							
Yes	5.97	<0.001	4.00	8.91	3.97	0.009	1.42	11.16

## Discussion

Our study evaluated the prevalence and predictors of stillbirths among 13,668 deliveries across 12 birthing facilities in Karachi, Pakistan, revealing an overall weighted stillbirth rate of 12.0 per 1,000 live births with notable variation across facility types and geographic locations. Nearly 50% of stillbirths occurred in preterm deliveries, with the highest weighted stillbirth rate observed in extreme preterm births (< 28 weeks, 429.1 per 1,000 live births). Key maternal health factors, such as ANC visits and lack of TT vaccination were also identified as significant predictors of stillbirth.

Our estimate of the weighted stillbirth rate of 12.0 per 1,000 births is lower than the reported national average of 30.9 per 1,000 total births [7]. We also found substantial variations in the weighted stillbirth rates based on location and facility characteristics. These findings are in line with global and local evidence from other low- and middle-income countries (LMICs) and Pakistan, which report lower than national level estimates for stillbirth rates and highlight variations across geographic locations and facility types, highlighting significant disparities in stillbirth outcomes [7, 12, 28, 29]. This observed deviation from national estimates may stem from several factors, including different study settings (urban vs. rural), underreporting, and challenges in identification of stillbirths by the hospital and healthcare staff, and exclusion of stillbirths occurring at home or enroute to healthcare facilities [12]. Despite this, neither district nor facility type, both assessed at the district level, emerge as significant predictors of stillbirth in our adjusted models. However, at the UC level, we found significantly higher stillbirths in facilities located in the polio-endemic areas (SHRUCs). Higher stillbirth rate in these areas could be because SHRUCs often face compounded challenges, including low maternal literacy, poor socioeconomic conditions, and limited healthcare access and information [28, 30, 31]. This, coupled by the lack of trust people have in the health system, further exacerbates the disparities in maternal and neonatal outcomes [32].

Our study findings revealed the substantial impact of gestational age on stillbirths, with the highest rate observed among extreme preterm deliveries (< 28 weeks) at 429.1 per 1,000 live births, and the lowest among at term deliveries (≥ 37 weeks) at 8.0 per 1,000 live births. This is consistent with findings from other global studies across multiple settings, where preterm delivery is consistently identified as major risk factors for stillbirths [33–36]. A possible explanation is that preterm birth increases the risk of stillbirth, primarily due to respiratory issues from an immature respiratory system, apnea (prolonged breathing pauses), heart problems, poor temperature regulation caused by insufficient body fat and a weakened immune system that makes infections more likely [34]. The high stillbirth rate among extreme preterm births raises important considerations for clinical care, as these deliveries are often associated with significant neonatal morbidity and mortality. It is worth noting that the stillbirth rate among preterm deliveries nationally may still be an underestimate as the current stillbirth reporting practices in Pakistan, (and many other LMICs), exclude stillbirths occurring before 28 weeks of gestation [14, 37–40]. Our findings emphasize the need for consistent application of the WHO’s recommended stillbirth definition, which includes stillbirths at any gestational age ≥22 weeks, to ensure more accurate data capture and informed intervention and policy development for timely and high-quality maternal care.

We also found key maternal health related factors which significantly increase the likelihood of stillbirths. Women who had not received TT vaccinations during pregnancy and who had received <8 ANC visits were at higher risk of stillbirth. Literature from other settings has signified the protective effect and safety of maternal immunization during pregnancy for improved birth outcomes and has been recommended by WHO as a key strategy for preventing vaccine-preventable diseases in newborns [14, 37–39, 41]. Adequate ANC visits (≥8) have been found to be associated with lower stillbirths across multiple settings [42, 43], highlighting the importance of comprehensive and continuous care across pregnancy. ANC contributes to stillbirth prevention by enabling timely detection and management of complications [44], and promoting institutional deliveries attended by skilled birth providers [45].

Household education was also associated with lower stillbirths in our study underscoring the importance of educated family members in improving maternal health and outcomes. Education contributes to improved health outcomes by enhancing communication within families and with healthcare providers, increasing household income and health-seeking capacity, fostering a greater sense of responsibility, and enabling timely recognition and response to danger signs [45, 46].

Our study had certain limitations. First, it was conducted only in selected birthing facilities in Karachi and focused solely on facility-based births (excluding home births) which limited generalizability. Additionally, the accurate identification of stillbirths among health staff was a challenge that has been documented and reported earlier in Pakistan [12]. Due to these challenges, we were unable to collect detailed data on the classification and nature of stillbirths (intrapartum vs. antepartum), which limits the depth of insights we can draw from our results. Additionally, to compute the stillbirth rates in our study, we relied on actual births within the study sample rather than estimated births for the broader setting due to the lack of reliable data. This limitation may affect the generalizability of our findings. Furthermore, we used the ≥ 22-week threshold to define stillbirths, consistent with the WHO/ICD-11 classification. While this enhances sensitivity and aligns with evolving global standards, it may limit comparability with national statistics and prior studies that use the ≥ 28-week threshold. The number of refusals was not recorded due to high patient volume and integration of registration into routine workflows. Based on field team observations, refusals were minimal. Lastly, although approximately one-third of participants were LTF—likely due to delivering outside of study facilities—we conducted a sensitivity analysis using IPW to adjust for differences in baseline characteristics. While this helps mitigate the risk of attrition bias, we acknowledge the potential for residual confounding due to unmeasured variables.

The findings from this study highlight several key areas where policy interventions could significantly reduce stillbirth rates. First, the higher stillbirth rates observed in SHRUC-based and government-operated facilities suggest a need for targeted investments in these settings to improve healthcare infrastructure, staffing, and quality of care. While substantial efforts have been directed toward polio eradication in SHRUCs, our findings underscore the need for a more integrated approach to health interventions. Focusing solely on polio may inadvertently overlook other pressing maternal and child health challenges, including stillbirths. To address this, we recommend that health policies in polio-endemic areas expand their scope to include comprehensive maternal health services. For example, maternal immunizations, such as tetanus toxoid vaccines, should be prioritized alongside routine polio campaigns to reduce infections that may contribute to adverse pregnancy outcomes. Additionally, ANC services must be enhanced in these areas, with a focus on improving both the quality and accessibility of care, a practice that has proven to be successful across multiple countries that have improved maternal and newborn healthcare over the last three decades [47]. Efforts should include better screening for maternal complications, early detection of risk factors, and timely management of conditions that could lead to stillbirths. Leveraging the existing infrastructure of polio programs, such as community outreach, vaccination campaigns, and healthcare worker networks — can facilitate the delivery of integrated maternal and child health services. Finally, given the high prevalence of preterm births and their association with stillbirths, policies that emphasize early identification of preterm labor and provide access to appropriate care and interventions could help reduce the risk of stillbirth, particularly in high-risk pregnancies. Future research is warranted to measure the prevalence of stillbirths at a wider-scale using real-time longitudinal data from electronic birth registries. Investigating the causal relationships between maternal health factors, immunization, and stillbirths, particularly in diverse settings, would offer valuable insights for targeted interventions.

## Conclusion

Our study found a stillbirth rate lower than the national average. However, the aggregate number masks underlying disparities between birthing facilities and geographic locations. Polio-endemic regions, in particular, emerged as hotspots for stillbirths, highlighting the need for targeted and integrated interventions. Strengthening maternal health services, through provision of high-quality antenatal care services including maternal immunization and efficient management of medical conditions during pregnancy remains critical to minimize the risk of stillbirths.

## Supplementary Information

Supplementary material 1.

## Data Availability

Data is available from the corresponding author upon reasonable request.

## Abbreviations

WHO: World health organization
SBR: Stillbirth rate
TT: Tetanus toxoid
LMICs: Low- and middle-income countries
PWBR: Pregnant women and birth registry
SHRUCs: Super high-risk union councils
SEIR: Sindh electronic immunization registry
ANC: Antenatal care
ODK: Open data kit
ICD: International classification of diseases
AOR: Adjusted odds ratio
