# Supplementary material for: Prevalence and risk factors of stillbirths among pregnant women from twelve high-volume birthing facilities of Karachi, Pakistan: a longitudinal cohort study
Source: BMC Pregnancy Childbirth. 2025 Dec 30;26:53. doi: 10.1186/s12884-025-08288-3 (PMC12805683; doi:10.1186/s12884-025-08288-3)
Supplement: Supplementary file 1 — Supplementary material 1. [file 12884_2025_8288_MOESM1_ESM.docx]

**Supplementary material**


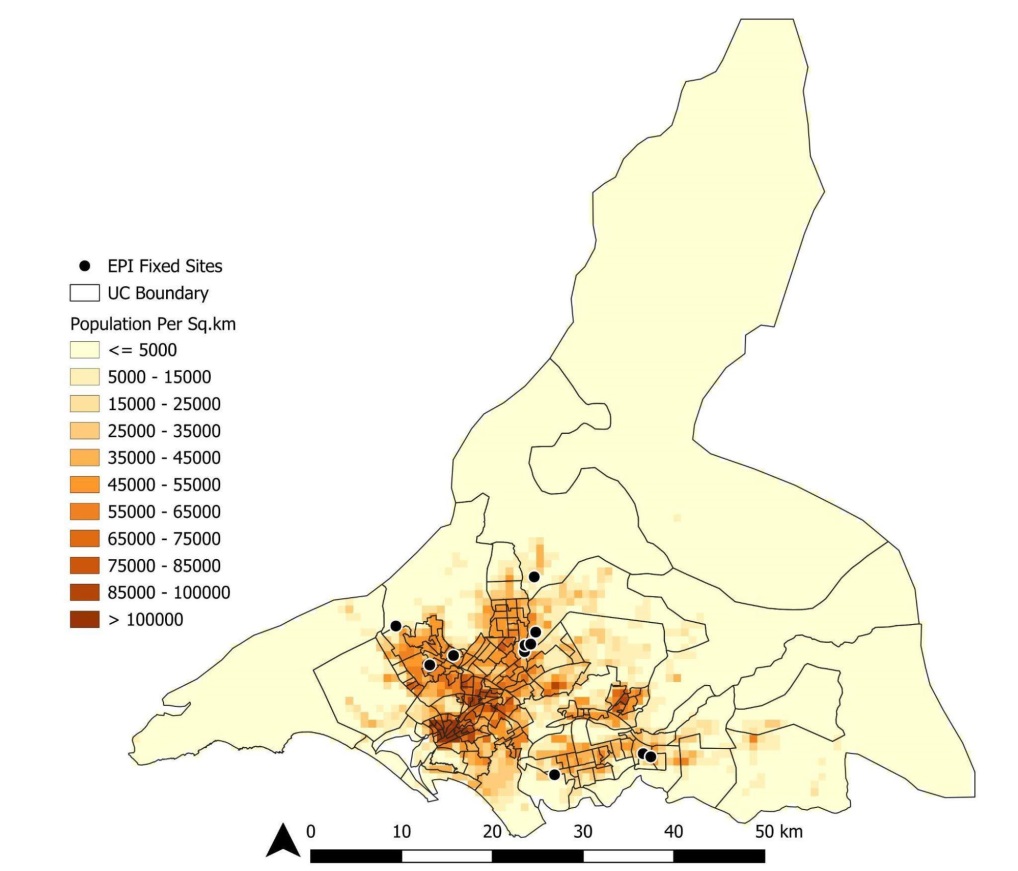
**Supplementary Figure 1:** Geographic Distribution of Study Sites

**Supplementary table 1.** Sociodemographic characteristics for women enrolled in 12 birthing facilities of Karachi, Sindh (n=21,523) by follow-up status (February 9, 2021 - January 1, 2022)

|  | **Followed-up (n=13,710)** |  | **Lost to follow-up (n=7,183)** |  | **Total (n=21,523)** |  | **P-value** |
| --- | --- | --- | --- | --- | --- | --- | --- |
| **Sociodemographic characteristics** | **Median** | **IQR** | **Median** | **IQR** | **Median** | **IQR** |  |
| Women age at enrollment (in years) | 25.4 | 22.3-30.4 | 25 | 22.2-30.4 | 25.4 | 22.3-30.4 | 0.966 |
| # of household members | 6 | 5-9 | 7 | 4-10 | 6 | 5-9 | <0.001 |
| # of children <5 years | 2 | 1-2 | 1 | 1-2 | 1 | 1-2 | <0.001 |
| # of elderly aged >65 years | 0 | 0-1 | 0 | 0-1 | 0 | 0-1 | - |
| # of children aged 5-18 years | 1 | 0-2 | 1 | 0-3 | 1 | 0-2 | - |
| Highest years of education received by household members | 9 | 0-10 | 0 | 0-1 | 8 | 0-10 | <0.001 |
| Weight of women (Kg) at last visit | 65 | 60-7 | 62 | 55-69 | 65 | 60-70 | <0.001 |
|  | **n** | **%** | **n** | **%** | **n** | **%** |  |
| **Districts of birthing facilities** |  |  |  |  |  |  |  |
| Karachi East | 580 | 20.1 | 2,300 | 79.9 | 2,880 | 100.0 | <0.001 |
| Karachi Kemari | 46 | 18.5 | 203 | 81.5 | 249 | 100.0 |  |
| Karachi Korangi | 2,988 | 95.1 | 154 | 4.9 | 3,142 | 100.0 |  |
| Karachi Malir | 2,007 | 75.2 | 661 | 24.8 | 2,668 | 100.0 |  |
| Karachi South | 4,192 | 63.7 | 2,388 | 36.3 | 6,580 | 100.0 |  |
| Karachi West | 3,897 | 64.9 | 2,107 | 35.1 | 6,004 | 100.0 |  |
| **Type of birthing facility** |  |  |  |  |  |  |  |
| Government | 10,217 | 65.0 | 5,510 | 35.0 | 15,727 | 100.0 | <0.001 |
| Private | 3,493 | 60.3 | 2,303 | 39.7 | 5,796 | 100.0 |  |
| Non-SHRUCs | 10,793 | 75.1 | 3,585 | 24.9 | 14,378 | 100.0 | <0.00 |
| SHRUCs | 2,917 | 40.8 | 4,228 | 59.2 | 7,145 | 100.0 |  |
| **Participant ethnicity** |  |  |  |  |  |  |  |
| Urdu speaking muhajirs | 6,825 | 74.9 | 2,290 | 25.1 | 9,115 | 100.0 | <0.001 |
| Sindhi | 823 | 67.1 | 404 | 32.9 | 1,227 | 100.0 |  |
| Punjabi | 695 | 65.8 | 361 | 34.2 | 1,056 | 100.0 |  |
| Pathan | 3,578 | 50.1 | 3,560 | 49.9 | 7,138 | 100.0 |  |
| Balochi | 607 | 64.8 | 330 | 35.2 | 937 | 100.0 |  |
| Others | 1,182 | 57.7 | 868 | 42.3 | 2,050 | 100.0 |  |
| **Participant education (in Years)** |  |  |  |  |  |  |  |
| 0 | 5,032 | 52.2 | 4,617 | 47.8 | 9,649 | 100.0 | <0.001 |
| 1-5 | 1,522 | 67.2 | 744 | 32.8 | 2,266 | 100.0 |  |
| 6-8 | 1,698 | 71.3 | 684 | 28.7 | 2,382 | 100.0 |  |
| 9-10 | 3,481 | 74.3 | 1,207 | 25.7 | 4,688 | 100.0 |  |
| >=11 | 1,977 | 77.9 | 561 | 22.1 | 2,538 | 100.0 |  |
| **Participant current occupation** |  |  |  |  |  |  |  |
| Housewife | 13,543 | 63.9 | 7,659 | 36.1 | 21,202 | 100.0 | <0.001 |
| Employed | 123 | 68.0 | 58 | 32.0 | 181 | 100.0 |  |
| Unemployed | 44 | 31.4 | 96 | 68.6 | 140 | 100.0 |  |
| **Monthly Household Income (PKR)** |  |  |  |  |  |  |  |
| <1,000 to 9,999 | 332 | 46.7 | 379 | 53.3 | 711 | 100.0 | <0.001 |
| 10,000 to 19,999 | 5,076 | 70.3 | 2,140 | 29.7 | 7,216 | 100.0 |  |
| 20,000 to 49,999 | 7,224 | 60.5 | 4,710 | 39.5 | 11,934 | 100.0 |  |
| >=50,000 | 1,078 | 64.9 | 584 | 35.1 | 1,662 | 100.0 |  |
| **History of any TT vaccine received** |  |  |  |  |  |  |  |
| No | 4,913 | 50.7 | 4,780 | 49.3 | 9,693 | 100.0 | <0.001 |
| Yes | 8,797 | 74.4 | 3,033 | 25.6 | 11,830 | 100.0 |  |
| **Use of any contraceptives^a^** |  |  |  |  |  |  |  |
| No | 12,125 | 62.4 | 7,306 | 37.6 | 19,431 | 100.0 | <0.001 |
| Yes | 1,585 | 75.8 | 507 | 24.2 | 2,092 | 100.0 |  |
| **Any pre-existing medical condition before ANC visit^b^** |  |  |  |  |  |  |  |
| No | 1,557 | 53.8 | 1,339 | 46.2 | 2,896 | 100.0 | <0.001 |
| Yes | 12,153 | 65.2 | 6,474 | 34.8 | 18,627 | 100.0 |  |
| **Any medications intake in pregnancy^d^** |  |  |  |  |  |  |  |
| No | 12,089 | 62.1 | 7,366 | 37.9 | 19,455 | 100.0 | <0.001 |
| Yes | 1,621 | 78.4 | 447 | 21.6 | 2,068 | 100.0 |  |
| **Use of any substances in pregnancy^f^** |  |  |  |  |  |  |  |
| No | 12,448 | 62.9 | 7,332 | 37.1 | 19,780 | 100.0 | <0.001 |
| Yes | 1,262 | 72.4 | 481 | 27.6 | 1,743 | 100.0 |  |
| **Body Mass Index (BMI) category^i^** |  |  |  |  |  |  |  |
| Underweight | 96 | 84.2 | 18 | 15.8 | 114 | 100.0 | <0.001 |
| Normal | 3,067 | 69.1 | 1,369 | 30.9 | 4,436 | 100.0 |  |
| Overweight | 4,001 | 79.6 | 1,024 | 20.4 | 5,025 | 100.0 |  |
| Obese | 2,053 | 81.1 | 477 | 18.9 | 2,530 | 100.0 |  |
| **Teenage Pregnancy (<20 years)** |  |  |  |  |  |  |  |
| No | 12,758 | 64.3 | 7,095 | 35.7 | 19,853 | 100.0 | <0.001 |
| Yes | 952 | 57.0 | 717 | 43.0 | 1,669 | 100.0 |  |

**Supplementary Table-2.** Unweighted and weighted univariable regression analysis for risk factors associated with stillbirths among women delivering at 12 selected birthing facilities in Karachi, Sindh (n=13,668) (February 9, 2021 - Jan 01, 2022)

|  | **Unweighted univariable regression** |  |  |  | **Weighted univariable regression** |  |  |  |
| --- | --- | --- | --- | --- | --- | --- | --- | --- |
|  | **OR** | **P-value** | **95% CI** |  | **HR** | **P-value** | **95% CI** |  |
| **Districts of birthing facilities** |  |  |  |  |  |  |  |  |
| Karachi West | 1 |  |  |  | 1 |  |  |  |
| Karachi East | 2.44 | 0.003 | 1.35 | 4.38 | 7.01 | 0.001 | 2.30 | 21.35 |
| Karachi Kemari | 7.43 | 0.001 | 2.4 | 23.02 | 6.81 | 0.063 | 0.90 | 51.52 |
| Karachi Korangi | 0.13 | <0.001 | 0.05 | 0.35 | 0.14 | 0.055 | 0.02 | 1.04 |
| Karachi Malir | 1 | 0.996 | 0.6 | 1.67 | 2.72 | 0.008 | 1.30 | 5.71 |
| Karachi South | 0.33 | <0.001 | 0.18 | 0.58 | 1.24 | 0.691 | 0.43 | 3.56 |
| **Type of birthing facility** |  |  |  |  |  |  |  |  |
| Private | 1 |  |  |  | 1.00 |  |  |  |
| Government | 1.67 | 0.051 | 1 | 2.8 | 2.57 | 0.058 | 0.97 | 6.81 |
| **Newborn delivered by** |  |  |  |  |  |  |  |  |
| Doctor | 1 |  |  |  | 1 |  |  |  |
| Midwife/lady health worker/relatives | 3.45 | <0.001 | 2 | 6 | 1.53 | 0.370 | 0.60 | 3.90 |
| **Birthing facilities in Super High-Risk Union Councils (SHRUCs) - Characterized by Polio endemic** |  |  |  |  |  |  |  |  |
| No | 1 |  |  |  | 1 |  |  |  |
| Yes | 2.86 | <0.001 | 1.93 | 4.23 | 5.73 | <0.001 | 2.63 | 12.51 |
| **Participant ethnicity** |  |  |  |  |  |  |  |  |
| Urdu speaking muhajirs | 1 |  |  |  |  |  |  |  |
| Sindhi | 1.27 | 0.574 | 0.55 | 2.9 | 3.84 | 0.094 | 0.80 | 18.53 |
| Punjabi | 0.81 | 0.698 | 0.27 | 2.4 | 0.91 | 0.904 | 0.21 | 3.98 |
| Pathan | 1.88 | 0.004 | 1.22 | 2.88 | 3.88 | 0.005 | 1.51 | 9.92 |
| Balochi | 0.92 | 0.883 | 0.31 | 2.75 | 1.02 | 0.984 | 0.14 | 7.65 |
| Others | 1.02 | 0.966 | 0.47 | 2.22 | 2.27 | 0.135 | 0.77 | 6.62 |
| **Participant education (in Years)** |  |  |  |  |  |  |  |  |
| >=11 | 1 |  |  |  | 1 |  |  |  |
| 0 | 2.83 | 0.008 | 1.31 | 6.08 | 4.27 | 0.004 | 1.59 | 11.46 |
| 1-5 | 1.65 | 0.305 | 0.63 | 4.32 | 1.09 | 0.892 | 0.33 | 3.60 |
| 6-8 | 2.11 | 0.102 | 0.86 | 5.16 | 1.26 | 0.685 | 0.41 | 3.91 |
| 9-10 | 1.56 | 0.3 | 0.67 | 3.6 | 0.65 | 0.426 | 0.23 | 1.87 |
| **Participant current occupation** |  |  |  |  |  |  |  |  |
| Clerical | 1 |  |  |  |  |  |  |  |
| Homemaker | 0.3 | 0.4 | 0.02 | 4.97 | - | - | - | - |
| Professional | 0.25 | 0.486 | 0 | 12.75 | 0.31 | 0.395 | 0.02 | 4.52 |
| Skilled Worker | 1.18 | 0.934 | 0.02 | 62.9 | 0.31 | 0.433 | 0.02 | 5.69 |
| Unemployed | 0.44 | 0.683 | 0.01 | 22.89 | 0.31 | 0.403 | 0.02 | 4.73 |
| Unskilled Worker | 2.05 | 0.724 | 0.04 | 111.62 | 0.31 | 0.420 | 0.02 | 5.23 |
| Women age (in years) | 1.01 | 0.473 | 0.98 | 1.05 | 1.04 | 0.258 | 0.97 | 1.11 |
| # of household members | 1.03 | 0.128 | 0.99 | 1.08 | 0.98 | 0.601 | 0.91 | 1.06 |
| # of children <5 years | 0.66 | <0.001 | 0.53 | 0.02 | 1.02 | 0.872 | 0.80 | 1.31 |
| # of children aged 5-18 years | 1.11 | 0.063 | 0.99 | 1.23 | 0.99 | 0.892 | 0.80 | 1.22 |
| # of elderly aged >65 years | 1.12 | 0.278 | 0.91 | 1.37 | 0.78 | 0.343 | 0.47 | 1.30 |
| Highest years of education received by household members | 0.96 | 0.019 | 0.92 | 0.99 | 0.85 | 0.000 | 0.78 | 0.92 |
| Weight of women (Kg) at last visit | 0.99 | 0.42 | 0.97 | 1.01 | 1.02 | 0.296 | 0.99 | 1.05 |
| **Monthly Household Income (PKR)** |  |  |  |  |  |  |  |  |
| >=50,000 | 1 |  |  |  | 1 |  |  |  |
| <1,000 to 9,999 | 3.28 | 0.033 | 1.1 | 9.81 | 1.73 | 0.582 | 0.25 | 12.07 |
| 10,000 to 19,999 | 1.16 | 0.731 | 0.5 | 2.68 | 1.92 | 0.304 | 0.55 | 6.66 |
| 20,000 to 49,999 | 1.27 | 0.563 | 0.56 | 2.87 | 4.29 | 0.036 | 1.10 | 16.76 |
| **Reported # of ANC visits** |  |  |  |  |  |  |  |  |
| >=8 | 1 |  |  |  | 1 |  |  |  |
| <8 | 1.76 | 0.012 | 1.13 | 2.75 | 4.20 | 0.001 | 1.85 | 9.57 |
| **History of any TT vaccine received** |  |  |  |  |  |  |  |  |
| Yes | 1 |  |  |  | 1 |  |  |  |
| No | 1.61 | 0.016 | 1.09 | 2.37 | 6.69 | <0.001 | 3.03 | 14.78 |
| **Use of any contraceptives^a^** |  |  |  |  |  |  |  |  |
| No | 1 |  |  |  | 1 |  |  |  |
| Yes | 1.36 | 0.265 | 0.79 | 2.34 | 0.36 | 0.052 | 0.13 | 1.01 |
| **Any pre-existing medical condition before ANC visit^b^** |  |  |  |  |  |  |  |  |
| No | 1 |  |  |  | 1 |  |  |  |
| Yes | 1.32 | 0.31 | 0.77 | 2.28 | 0.30 | 0.014 | 0.12 | 0.78 |
| **Any medical condition during ANC visit^c^** |  |  |  |  |  |  |  |  |
| No |  |  |  |  | 1 |  |  |  |
| Yes | 1.85 | 0.002 | 1.25 | 2.75 | 1.50 | 0.463 | 0.51 | 4.40 |
| **Any medications intake in pregnancy^d^** |  |  |  |  |  |  |  |  |
| No | 1 |  |  |  | 1 |  |  |  |
| Yes | 1.38 | 0.12 | 0.92 | 2.06 | 0.45 | 0.166 | 0.15 | 1.39 |
| **Any supplement intake in pregnancy^e^** |  |  |  |  |  |  |  |  |
| No | 1 |  |  |  | 1 |  |  |  |
| Yes | 0.77 | 0.503 | 0.37 | 1.63 | 1.41 | 0.748 | 0.17 | 11.71 |
| **Practicing any precautions in pregnancy^f^** |  |  |  |  |  |  |  |  |
| No | 1 |  |  |  | 1 |  |  |  |
| Yes | 0.91 | 0.686 | 0.59 | 1.42 | 0.44 | 0.131 | 0.15 | 1.27 |
| **Use of any substances in pregnancy^g^** |  |  |  |  |  |  |  |  |
| No | 1 |  |  |  | 1 |  |  |  |
| Yes | 0.77 | 0.503 | 0.37 | 1.63 | 0.24 | 0.011 | 0.08 | 0.72 |
| **Body Mass Index (BMI) category^j^** |  |  |  |  |  |  |  |  |
| Obese | 1 |  |  |  | 1 |  |  |  |
| Underweight | 2.81 | 0.237 | 0.51 | 15.62 | - | - | - | - |
| Normal | 1.14 | 0.726 | 0.55 | 2.37 | 1.18 | 0.827 | 0.27 | 5.22 |
| Overweight | 1.41 | 0.321 | 0.72 | 2.77 | 2.18 | 0.177 | 0.70 | 6.75 |
| **Preterm delivery (<37 weeks)** |  |  |  |  |  |  |  |  |
| No | 1 |  |  |  | 1 |  |  |  |
| Yes | 5.14 | <0.001 | 3.47 | 7.62 | 4.52 | 0.005 | 1.57 | 13.04 |
| **Teenage Pregnancy (<20 years)** |  |  |  |  |  |  |  |  |
| No | 1 |  |  |  | 1 |  |  |  |
| Yes | 0.47 | 0.165 | 0.16 | 1.36 | 0.11 | 0.031 | 0.01 | 0.81 |
| **Use of Folic Acid** |  |  |  |  |  |  |  |  |
| No | 1 |  |  |  | 1 |  |  |  |
| Yes | 0.93 | 0.76 | 0.59 | 1.48 | 0.59 | 0.377 | 0.19 | 1.89 |
| **Use of Vitamin D** |  |  |  |  |  |  |  |  |
| No | 1 |  |  |  | 1 |  |  |  |
| Yes | 1.06 | 0.788 | 0.71 | 1.57 | 1.37 | 0.504 | 0.54 | 3.46 |
| **Use of Calcium** |  |  |  |  |  |  |  |  |
| No | 1 |  |  |  | 1 |  |  |  |
| Yes | 1.00 | 0.988 | 0.66 | 1.5 | 0.46 | 0.158 | 0.16 | 1.35 |
| **Use of Iron** |  |  |  |  |  |  |  |  |
| No | 1 |  |  |  | 1 |  |  |  |
| Yes | 0.7 | 0.129 | 0.45 | 1.11 | 0.10 | <0.001 | 0.03 | 0.34 |
| **Use of Magnesium** |  |  |  |  |  |  |  |  |
| No | 1 |  |  |  | 1 |  |  |  |
| Yes | 1.26 | 0.505 | 0.64 | 2.46 | 4.34 | 0.005 | 1.56 | 12.10 |
| **Anemic (pre or during pregnancy)** |  |  |  |  |  |  |  |  |
| No | 1 |  |  |  | 1 |  |  |  |
| Yes | 0.82 | 0.575 | 0.4 | 1.65 | 0.14 | 0.002 | 0.04 | 0.50 |
| **Hypertension (pre- or during pregnancy)** |  |  |  |  |  |  |  |  |
| No | 1 |  |  |  | 1 |  |  |  |
| Yes | 2.34 | 0.037 | 1.05 | 5.21 | 1.08 | 0.888 | 0.38 | 3.04 |

1. Contraceptives include condoms, implants, injections, oral pills, and surgical procedures
2. Pre-existing medical condition before ANC visit includes any one of the key reported conditions anemia, hypertension, obesity, diabetes, chronic heart/liver/kidney diseases among others
3. Medical conditions during ANC visit include any one of the key reported conditions including fatigue, reflux, lower back/neck pain, nausea/vomiting pelvic pain, and anemia among others.
4. Medication intake in pregnancy includes the use of any one of the key medications including painkillers, fever medicines, antibiotics, and anti-allergies, among others.
5. Supplements intake in pregnancy includes the use of any one of the key supplements including folic acid, iron/vitamin D, and calcium, among others.
6. Practicing any precautions in pregnancy includes any one of the precautions including washing hands more frequently than usual, washing food before eating, avoiding raw food, resting more than usual, and avoiding contact with animals among others
7. Substance use in pregnancy includes any one of the substances including beetal nuts, prescription drugs for medical/non-medical use, tobacco, and alcohol.
8. Postpartum condition includes any of the conditions including admission to intensive care unit, pelvic floor disorder, postpartum hemorrhage, among others.
